# Engineering Adhesive Hydrogels for Hemostasis and Vascular Repair

**DOI:** 10.3390/polym17070959

**Published:** 2025-04-01

**Authors:** Juya Jeon, Shri Venkatesh Subramani, Kok Zhi Lee, Santiago Elizondo-Benedetto, Mohamed Adel Zayed, Fuzhong Zhang

**Affiliations:** 1 Department of Energy, Environmental & Chemical Engineering, Washington University in St. Louis, Saint Louis, MO 63130, USA; jjuya@wustl.edu (J.J.); s.shrivenkatesh@wustl.edu (S.V.S.); kokzhi@wustl.edu (K.Z.L.); 2 Section of Vascular Surgery, Department of Surgery, Washington University School of Medicine in St. Louis, Saint Louis, MO 63130, USA; esantiago@wustl.edu (S.E.-B.); zayedm@wustl.edu (M.A.Z.); 3 Division of Surgical Sciences, Department of Surgery, Washington University School of Medicine in St. Louis, Saint Louis, MO 63130, USA; 4 Department of Biomedical Engineering, Washington University in St. Louis, Saint Louis, MO 63130, USA; 5 Cardiovascular Research Innovation in Surgery & Engineering Center, Department of Surgery, Washington University School of Medicine in St. Louis, Saint Louis, MO 63130, USA; 6 Institute of Materials Science and Engineering, Washington University in St. Louis, Saint Louis, MO 63130, USA; 7 Division of Biological & Biomedical Sciences, Washington University in St. Louis, Saint Louis, MO 63130, USA

**Keywords:** adhesive hydrogel, hemostasis, surgical adhesives, vascular repair, protein polymers

## Abstract

Adhesive hydrogels with tunable mechanical properties and strong adhesion to wet, dynamic tissues have emerged as promising materials for tissue repair, with potential applications in wound closure, hemorrhage control, and surgical adhesives. This review highlights the key design principles, material classifications, and recent advances in adhesive hydrogels designed for vascular repair. The limitations of existing adhesive hydrogels, including insufficient mechanical durability, suboptimal biocompatibility, and challenges in targeted delivery, are critically evaluated. Furthermore, innovative strategies—such as incorporating self-healing capabilities, developing stimuli-responsive systems, integrating functional nanocomposites, and employing advanced fabrication techniques like 3D bioprinting—are discussed to enhance adhesion, mechanical stability, and vascular tissue regeneration. While significant progress has been made, further research and optimization are necessary to advance these materials toward clinical translation, offering a versatile and minimally invasive alternative to traditional vascular repair techniques.

## 1. Introduction

Bleeding and hemorrhagic shock have a significant impact on patient morbidity and mortality, with uncontrolled bleeding being a leading cause of mortality worldwide, responsible for over two million deaths annually [1,2]. Trauma-related hemorrhage alone has a reported fatality rate of approximately 30–40% [3], while hemorrhage complications occur in nearly 7% of cardiac surgeries and percutaneous coronary interventions where patient underlying morbidity is already high [4]. Additionally, emergency coronary artery bypass graft surgeries following percutaneous coronary interventions exhibit mortality rates between 7.4% and 21% [5]. These high fatality rates stem from the challenges associated with managing and maintaining appropriate hemostasis to prevent hemodynamic compromise and progression to shock [6].

Current techniques for treating surgical and trauma-related bleeding, such as compression with absorbable textiles, often fail to effectively stop massive hemorrhage [7]. To address this, surgical toolkits often incorporate hemostatic gauzes, tissue adhesives, and sealants designed to promote blood coagulation and adhere to the adjacent tissue surfaces. Beyond wound management, hemostatic materials are increasingly explored for endovascular embolization, wherein biomaterials occlude blood vessels to prevent tissue rupture [8]. The current standard, coil embolization, relies on inserting a coil-shaped metal wire to induce clot formation; however, its efficacy is reduced in patients with coagulopathic diseases [8]. Therefore, the development of advanced biomaterials is crucial for improving effective hemorrhage management strategies.

**Figure 1:**
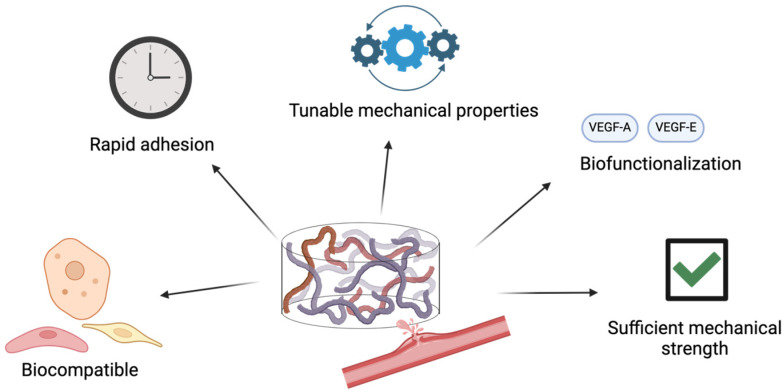
Engineering adhesive hydrogels with desirable properties for vascular repair.

Adhesive hydrogels have emerged as a particularly promising class of biomaterials for hemorrhage management and wound repair. Adhesive hydrogels not only absorb blood locally but also form strong molecular interactions with surrounding tissues, enabling rapid hemostasis and wound sealing. Among various tissue repair scenarios, vascular repair represents some of the greatest challenges due to the dynamic mechanical environment and the need for rapid hemostasis under high-pressure conditions. To be effective for vascular repair, adhesive hydrogels must meet several key criteria (Figure 1). **(1) Biocompatibility**—The material must be compatible with vascular and surrounding connective tissues. Incompatible materials, such as cyanoacylate-based sealants, have been shown to induce inflammation and tissue necrosis in vivo [9,10]. **(2) Rapid Adhesion**—Since hemorrhagic events are time-sensitive and life-threatening, the adhesive must act rapidly to stop bleeding and prevent fatal blood loss. **(3) Tunable Mechanical Properties**—The adhesive’s mechanical properties should be adjustable to accommodate different parts of the body with variable vascular structures. For example, arteries generally have a higher modulus and burst pressure than veins, thus requiring tailored performance for optimal sealing [11]. **(4) Biofunctionalization**—The adhesive should support the integration of bioactive molecules, such as vascular endothelial growth factors, to promote wound healing and accelerate recovery [12]. **(5) Sufficient Mechanical Strength**—To function effectively, the adhesive must withstand burst pressures of systolic blood exceeding 180 mmHg, the upper limit of human blood pressure, ensuring durability under physiological conditions [13]. Together, these criteria define the performance requirements for next-generation vascular adhesive hydrogels. Meanwhile, some real-time monitoring systems in hydrogels have been developed to monitor the wound healing process. However, these systems are limited to hydrogels applied on the surface of the body. Developing a deep-tissue monitoring system would be highly valuable for vascular adhesives, especially for fine-tuning the release of growth factors to accelerate the healing process [14,15].

While several engineered adhesive hydrogels have successfully met some of these criteria, none have yet fully satisfied all the requirements for optimal vascular repair applications. In this review, we explore natural and synthetic vascular adhesives, discuss vascular hydrogels for vascular applications, and highlight current challenges and future directions for vascular sealants.

## 2. Different Vascular Adhesives

**Figure 2:**
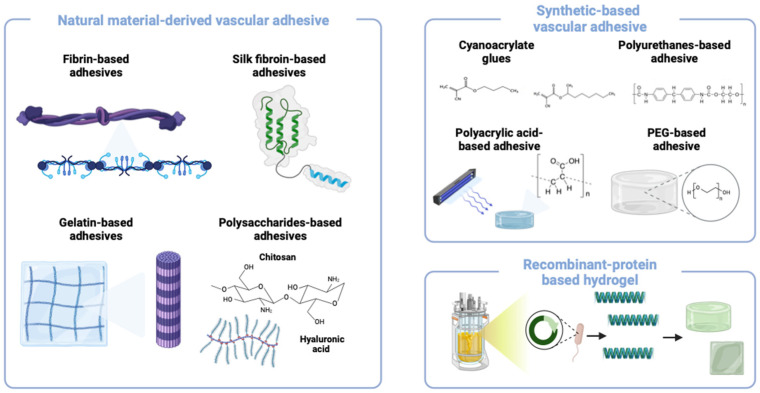
Different materials used for vascular adhesives.

Vascular adhesives can be categorized based on their composition and adhesion mechanisms, including natural polymer-based (e.g., fibrin, gelatin), synthetic polymer-based (e.g., cyanoacrylates, polyurethane), and hybrid adhesives that combine natural and synthetic components (Figure 2). In this section, we discuss these materials based on their biocompatibility, biodegradability, and adhesion performance in biological tissues [16,17,18]. Adhesives that lack hemostatic properties, exhibit cytotoxicity, or have not been validated on biological tissues are excluded from this review (Table 1).

### 2.1. Natural Material-Derived Vascular Adhesive

Some natural material-derived vascular adhesives offer good biocompatibility, biodegradability, and inherent hemostatic properties, supporting tissue integration and promoting healing. However, these adhesives typically suffer from weak adhesion, limited mechanical stability, and batch-to-batch variability, all of which hinder their broader application in vascular repair. Additionally, immunogenic risks and complex processing steps further complicate their clinical translation [33,34,35]. This section highlights the strategies researchers have developed to address these challenges, while also discussing the remaining limitations of natural material-based vascular adhesives.

#### 2.1.1. Fibrin-Based Adhesives

Fibrin-based hydrogels are widely used in tissue engineering and regenerative medicine due to their biocompatibility, potential for autologous production, low cytotoxicity, and functions in wound healing and clot formation [36]. Fibrin glue, derived from the body’s natural coagulation system, consists of two components. The first component contains concentrated fibrinogen, and the other component comprises thrombin. Upon mixing, thrombin catalyzes the conversion of fibrinogen into fibrin, which polymerizes into a stable clot within 30 s. Clinically, fibrin glue has been used for over three decades in cardiovascular surgery to control bleeding and is routinely applied as a sealant for needle hole closure in vascular graft anastomoses [19,20,37].

Despite its clinical success, fibrin glue has notable limitations, including low adhesive strength, poor durability, and susceptibility to infection. These shortcomings largely stem from the low fibrinogen concentrations used in most formulations, which are intended to prevent the formation of dense polymer networks that could otherwise inhibit cell infiltration and migration. Therefore, the low-density molecular architecture of fibrin, together with the limited fibrin–fibrin interactions and crosslinking, leads to the formation of loosely interconnected hydrogels, inherently limiting their cohesive strength and mechanical durability [19]. Also, at such low concentrations, the resulting material becomes extremely soft and lacks the mechanical strength required for effective adhesion and tissue sealing. Additionally, these overly soft gels are impractical for clinical handling due to their fragility and inability to withstand manipulation [38]. Additionally, many fibrin-based adhesives exhibit rapid activation, limiting the ability for fine adjustments or the repositioning of surgical devices. Furthermore, fibrin glues are constrained with regard to their use in intervascular applications due to the risk of embolization, rendering them ineffective in vascular surgery [18].

To overcome these limitations, researchers have explored the incorporation of additional materials into fibrin formulations to enhance both adhesive strength and mechanical performance. One such approach involved integrating fibrin into collagen carriers to develop ready-to-use fibrinogen-coated collagen patches. While the collagen matrix improved the overall mechanical integrity of the patch, its adhesive strength to biological tissues in the presence of blood only reached approximately 3–5 kPa—significantly lower than the physiological blood pressure required for effective vascular sealing [39].

#### 2.1.2. Silk Fibroin-Based Adhesives

Silk fibroin (SF), a natural polymer derived from the silkworm Bombyx mori, has been extensively explored for biomedical applications due to its desirable mechanical properties and biocompatibility. Structurally, SF consists of a 26 kDa light chain and a 390 kDa heavy chain linked by a disulfide bond. Its repetitive Gly–Ala–Gly–Ala–Gly–Ser sequences promote β-sheet formation through self-assembly, forming strong intermolecular hydrogen bonding, therefore contributing to its robust mechanical properties [40]. As a biomaterial, SF offers excellent biocompatibility, low immunogenicity, tunable biodegradability, and favorable water permeability, making it well suited for a range of tissue engineering applications [21,22,39].

Through various fabrication techniques, SF can be processed into fibers, films, sponges, hydrogels, and sealants, demonstrating versatility for biomedical use. However, SF-based adhesives inherently lack functional groups capable of forming strong covalent or non-covalent bonds with tissue surfaces [22,41]. As a result, they typically exhibit poor adhesion to wet, bleeding tissues, particularly under dynamic conditions such as pulsatile blood flow or heart contractions, limiting their effectiveness for vascular repair [21]. To address this challenge, Bai et al. incorporated tannic acid into an SF-based hydrogel, enabling rapid sealing of severely bleeding tissues under wet and highly dynamic conditions in rat models. Although this formulation exhibited relatively long gelation times, it demonstrated excellent biocompatibility, biodegradability, and antibacterial properties [22]. Similarly, Kim et al. developed a methacrylate-modified SF sealant (Sil-MAS) with enhanced mechanical properties, achieving an ultimate tensile stress of 32.9 kPa and withstanding burst pressures in the range of 90–140 mmHg, approaching physiological arterial pressures. Sil-MAS demonstrated effective hemostatic and adhesive performance in in vivo tests on rat skin, liver, and blood vessels, significantly reducing wound healing time compared to commercially available products [42]. Despite these promising advances, the need for extensive silk fibroin purification, functionalization, and processing presents challenges for large-scale production, requiring further optimization to enhance clinical feasibility [43].

#### 2.1.3. Gelatin-Based Adhesives

Gelatin sponges, such as Gelfoam^®^ from Pfizer (New York, NY, USA), are commercially available and widely used for controlling surgical bleeding due to their excellent biocompatibility and hemostatic properties. More recently, gelatin nanofiber sponges fabricated via electrospinning have been proposed for treating arterial injuries, demonstrating excellent hemostatic performance compared to conventional medical gauzes and traditional gelatin sponges [23,44]. In addition to sponges, gelatin methacryloyl (GelMA) hydrogels have gained attention as promising candidates for physical hemostasis in vivo, thanks to their tunable mechanical properties and strong adhesion capabilities. Zhou et al. developed a hybrid adhesive by combining GelMA with hyaluronic acid (HA) modified with N-(2-aminoethyl)-4-(4-(hydroxymethyl)-2-methoxy-5-nitrosophenoxy) butanamide (NB), which enhances adhesion and hemostasis [23]. The NB-functionalized HA enables on-demand aldehyde generation upon UV irradiation, facilitating Schiff base formation with tissue surfaces. This mechanism promotes strong adhesion, enabling the hydrogel to form robust bonds with vascular tissues and withstand blood pressures up to 250 mmHg. This strong adhesive hydrogel effectively sealed both ~6 mm diameter cardiac wounds and 4–5 mm long carotid artery incisions, demonstrating its potential for vascular repair [23].

#### 2.1.4. Polysaccharides-Based Adhesives

Polysaccharide-based hydrogels, particularly those derived from chitosan and hyaluronic acid (HA), have been extensively studied for vascular applications due to their inherent biocompatibility, biodegradability, and hemostatic properties.

Chitosan, derived from chitin, also possesses antibacterial properties, making it particularly useful for wound healing in infection-prone environments. However, chitosan’s limited water absorbability restricts its practical application as a hemostatic adhesive [24,45,46]. To overcome this limitation, Song et al. developed a bio-hydrogel by combining 3-(3,4-dihydroxyphenyl) propionic acid-modified chitosan with p-hydroxybenzaldehyde-modified PEG sebacate, avoiding the use of toxic crosslinking reagents [45]. The resulting hydrogel exhibited impressive stretchability (up to 780%) and exceptional blood absorbability (1300% ± 50%). Moreover, the hydrogel demonstrated strong adhesion (~68.5 kPa) to porcine skin and successfully sealed arterial injuries in rat models while also providing antibacterial protection, enhancing its potential for vascular applications [45].

Hyaluronic acid (HA)-based hydrogels also show promise for vascular repair due to HA’s excellent hydration properties and its ability to support cell migration, tissue regeneration, and angiogenesis [25]. HA-methacrylate (HA-ME) can be synthesized via transesterification and photopolymerized under UV irradiation. However, photopolymerization often generates free radicals that can compromise cell viability, particularly for sensitive cell types. Furthermore, crosslinked HA hydrogel lacks sufficient tissue adhesion for effective cell transplantation [25,47,48]. To overcome these challenges, Shin et al. employed mussel-inspired catechol chemistry, utilizing dopamine polymerization under alkaline conditions to create HA-catechol (HA-CA) hydrogels [26]. This UV-free approach improved biocompatibility and significantly enhanced tissue adhesion, achieving an adhesion strength of 47 ± 36 kPa on rat heart tissue, demonstrating its potential for vascular sealing and tissue regeneration [26].

Further advancing polysaccharide-based adhesives, Yuan et al. developed a multifunctional hydrogel utilizing a dual crosslinking strategy [49]. This system combined Schiff base crosslinking between catechol-modified oxidized hyaluronic acid (OD) and aminated gelatin (AG), with coordination crosslinking between OD and Fe^3^⁺ ions. Upon injection into a wound site, the prepolymer solution rapidly gelled upon contact with blood, forming a tight adhesive seal capable of stopping bleeding within seconds. In in vivo rat femoral vein puncture models, the hydrogel provided instant hemostasis, firmly adhered to the bleeding vessels, and effectively sealed pinhole injuries caused by needle removal [49].

### 2.2. Synthetic-Based Vascular Adhesive

In addition to natural polymer-based adhesives, synthetic polymer hydrogels offer precise control over physical and chemical properties, including swelling behavior, mechanical strength, and surface charge. This section reviews recent developments in synthetic polymer-based vascular adhesives, focusing on materials such as polyurethanes, polyacrylic acid, and polyethylene glycol (PEG) [2,18,50]. Cyanoacrylate-based adhesives, despite their strong adhesion and stability, are excluded from this review due to their lack of elasticity and known cytotoxicity.

#### 2.2.1. Polyurethane-Based Adhesive

Polyurethane (PU)-based adhesives have gained attention for vascular applications due to their mechanical properties, which can tuned by controlling urethane linkage formation [27]. However, these materials may suffer from biocompatibility issues, as residual isocyanates or crosslinking agents can trigger chronic inflammation or immune reactions. When directly exposed to blood or vascular tissues, these molecular residues pose risks, highlighting the need for extensive purification and stabilization during synthesis [50]. Schulten et al. developed a PU adhesive that polymerizes rapidly when mixed with an amino-functional aspartic acid ester curing agent. This PU adhesive demonstrated sufficient burst pressure resistance and tensile strength to facilitate sutureless microsurgical anastomoses in ex vivo vascular models [27]. However, the successful application of this adhesive relied heavily on precise vessel alignment during application, which could be challenging under realistic surgical conditions. Furthermore, long-term studies investigating the adhesive’s degradation behavior and biocompatibility in in vivo vascular environments remain necessary to fully evaluate its clinical potential.

#### 2.2.2. Polyacrylic Acid-Based Adhesive

Polyacrylic acid (PAA)-based hydrogels have also been explored for vascular applications, primarily due to their high water retention capacity and ability to swell in response to pH changes—properties that make them particularly suitable for moist environments such as blood vessels. However, PAA-based adhesives generally lack strong covalent bonding with tissue surfaces, resulting in relatively weak adhesion, particularly after the initial swelling phase [29,51,52]. To enhance adhesion and flexibility, Ito et al. developed a hybrid adhesive hydrogel using PAA and poly (vinylpyrrolidone) (PVP) through a solid/solution interface complexation method [30]. In this system, the carboxyl groups of PAA remained partially uncomplexed, preserving PAA’s natural affinity for biological tissues. The hydrogel effectively adhered to moist beef tissue, demonstrating its potential for clinical applications. In an in vivo rat jugular vein incision model, the PAA/PVP adhesive film successfully achieved complete hemostasis within 15 min of application [30]. However, further studies are needed to evaluate the long-term performance and stability of PAA-based adhesives after swelling in dynamic vascular environments.

#### 2.2.3. Polyethylene Glycol (PEG)-Based Adhesive

Polyethylene glycol (PEG)-based materials are widely used in biomedical applications due to their high water solubility, biocompatibility, and tunable mechanical properties [17]. One clinically approved PEG-based sealant, Coseal^®^ (Deerfield, IL, USA), has been successfully used in vascular surgery to prevent anastomotic leaks. Upon application, PEG in sodium phosphate/sodium carbonate solution reacts with PEG in dilute hydrogen chloride solution, forming a crosslinked hydrogel network. This synthetic PEG hydrogel also forms covalent bonds with amine groups on tissue surfaces, enhancing adhesion strength [16,53,54].

To further improve tissue adhesion and promote endothelial healing, researchers have explored hybrid formulations incorporating gelatin, catechol, and dendrimeric components [17]. Wang et al. developed a bio-inspired adhesive combining amino-gelatin and tetra-armed PEG succinimidyl succinate, mimicking the extracellular matrix [28]. This adhesive exhibited high toughness, rapid self-gelation, and controlled degradation when exposed to a cysteamine solution. In in vivo studies, the adhesive effectively sealed severe hemorrhages caused by aortic rupture and cardiac puncture injuries, with postoperative analysis confirming robust sealing, excellent blood compatibility, and accelerated healing [28]. Ongoing studies continue to optimize crosslinking chemistry for PEG-based adhesives to balance adhesion strength and tissue compatibility [55,56].

### 2.3. Recombinant-Protein Based Hydrogel

Adhesive hydrogels made from microbially produced recombinant proteins offer excellent biocompatibility, bio-absorbability, and tunable sequences, allowing both adhesive and mechanical properties to be precisely controlled [31,32,57,58]. These properties make recombinant protein-based hydrogels highly attractive for biomedical applications, including vascular repair [59,60].

One notable example is recombinant mussel foot proteins (Mfp), which display strong underwater adhesion to a wide range of surfaces, making them a promising base material for tissue adhesives [61,62,63]. However, Mfp proteins are intrinsically disordered and exhibit strong self-interaction, leading to significant protein aggregation under most conditions. This aggregation makes it difficult to form stable hydrogels directly from Mfp proteins. In contrast, silk and amyloid proteins provide excellent mechanical support through their ability to self-assemble into stable, fibrous structures [31,64,65,66,67]. To leverage the complementary properties of these proteins, recombinant hybrid proteins incorporating repetitive sequences from silk, amyloid, and Mfp domains—referred to as SAM proteins—have been developed [31,32]. These SAM proteins can be processed into hydrogels, where the repetitive amyloid and silk segments self-assemble into needle-like nanostructures. This self-assembly process effectively separates the adhesive Mfp fragments, allowing them to remain available for surface bonding. The resulting SAM hydrogels exhibited exceptionally strong underwater adhesion to porcine skin and other biological tissues, with adhesive strength reaching 416 ± 20 kPa [32]. Furthermore, the mechanical strength, modulus, toughness, and adhesive performance of SAM hydrogels can be fine-tuned by adjusting the length and composition of the individual protein segments, enabling the design of adhesives with tailored properties for specific applications [32].

A long-standing challenge associated with recombinant protein-based materials has been their relatively low production yield, which limited scalability for clinical applications. However, advances in high-cell-density fermentation have significantly improved production efficiency, with protein yields reaching 8–30 g/L [68,69]. These improvements enhance the feasibility of recombinant protein-based adhesives for future biomedical and vascular repair applications.

## 3. Applications and Outlook of Vascular Adhesive Hydrogels

### 3.1. Applications of Adhesive Hydrogels in Vascular Surgery and Repair

#### 3.1.1. Hemostasis

**Figure 3:**
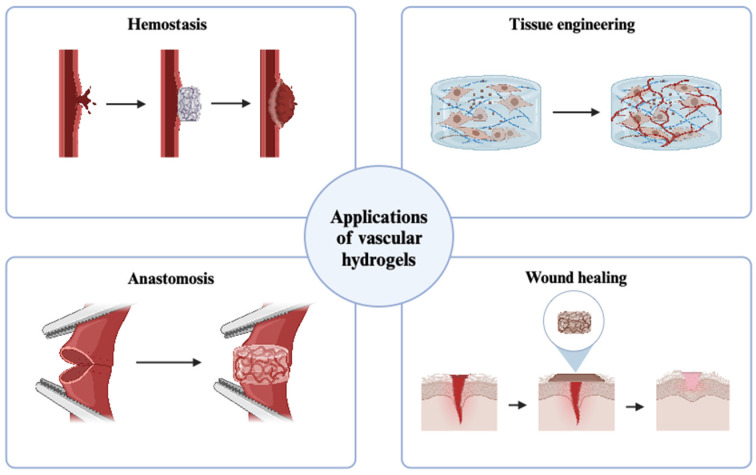
Applications of vascular hydrogels.

One of the primary functions for adhesive hydrogels in vascular repair is in hemostasis, where they function as potent blood clotting agents [70]. These materials create a physical barrier over injured blood vessels, preventing further blood loss while promoting superficial blood clot formation. Their strong adhesive properties allow them to form stable seals on wet and pulsating tissues, making them valuable in emergency trauma care and in operative settings. An ideal vascular adhesive hydrogel should possess the following key properties and should (i) rapidly stop hemorrhage by gelling within 5–10 s and promote wound healing through strong adhesion to wet tissues; (ii) maintain sufficient mechanical strength to withstand physiological blood pressure; (iii) be composed of biocompatible materials, with a hemolysis rate of ≤5%; and (iv) fully degrade within a timeframe aligned with the natural wound healing process [71]. Multiple hydrogels have the potential to meet these targets for hemostasis purposes, including silk [22], gelatin [72], keratin [73], chitosan [74], alginate [75], hyaluronic acid [76], and polyethylene glycol [77]. These materials are endowed with strong adhesion properties under wet conditions by employing mussel-inspired compounds such as dopamine and dihydroxyphenylalanine (DOPA), which contain catechol groups, capable of adhering to wet surfaces [78]. Common approaches to introduce catechol groups include the enzymatic conversion of tyrosine residues in protein, introduction of polydopamine functionalized nanomaterials, conjugation of choline-containing compounds, and introduction of catechin nanoparticles [73,79,80,81]. Some hydrogel formulations also incorporate hemostatic agents, such as thrombin, fibrinogen, or bioactive peptides to accelerate coagulation and improve wound closure efficiency (Figure 3).

#### 3.1.2. Vascular Anastomosis

Adhesive hydrogels also play a critical role in vascular anastomosis, the process of surgically connecting blood vessels. While traditional monofilament sutures are known to be effective, they can be associated with longer operative times, technical complications, suture line leakage, and narrowing and thrombosis of blood vessels. In contrast, vascular adhesive hydrogels offer a minimally invasive alternative by forming leak-proof and flexible seals on the external surface of the vascular structure and at anastomosis sites. Rapid management in this regard would reduce surgical trauma, reduce the operative times, and potentially improve patient clinical outcomes. For example, Muñoz Taboada et al. [82] designed a novel adhesive hydrogel for rapid and efficient vascular anastomosis. The hydrogel, composed of oxidized alginate, oxidized dextran, and polyamidoamine dendrimer amine, forms a dual network that interacts electrostatically and covalently with tissue surfaces. This formulation ensured strong adhesion, allowing the hydrogel to instantly seal vascular anastomosis sites and withstand high blood pressure readings (~300 mmHg). Moreover, when compared to commercial alternatives, such as Tisseel (Deerfield, IL, USA) and BioGlue (Kennesaw, GA, USA), this hydrogel demonstrated superior hemostatic efficiency and durability, significantly reducing the risk of leakage and thrombosis in surgical procedures. Lastly, in vivo animal model studies confirmed its efficacy in promoting healing while minimizing inflammatory responses [82], highlighting its potential as a minimally invasive, efficient, and reliable alternative to conventional sutures and adhesives.

#### 3.1.3. Wound Healing

Adhesive hydrogels contribute significantly to wound healing by serving as scaffolds that support endothelial cell migration and angiogenesis during vascular repair [83]. Their ability to retain moisture creates an optimal environment for tissue regeneration, preventing desiccation and facilitating cellular interactions. Some formulations are enriched with growth factors, such as vascular endothelial growth factor (VEGF) and platelet-derived growth factor (PDGF), enhancing angiogenesis and accelerating healing in chronic wounds and burns. Li et al. developed silica-based nanocomposite hydrogel scaffolds with enhanced angiogenic properties to promote diabetic wound healing and skin repair [84,85,86]. Chronic diabetic wounds pose significant treatment challenges due to poor perfusion, higher infection risks, and impaired healing. The silica-based nanocomposite hydrogel provides a moist wound environment while releasing bioactive silica, which enhances endothelial cell proliferation and angiogenesis. Additionally, the nanocomposites exhibit strong antibacterial activity against common wound pathogens, reducing infection risks. The hydrogel also supports cell adhesion, migration, and collagen deposition, further accelerating tissue regeneration. Both in vitro and in vivo studies demonstrated the hydrogel’s ability to promote neovascularization, re-epithelialization, and extracellular matrix remodeling, significantly improving wound closure rates in diabetic models.

#### 3.1.4. Tissue Regeneration

Hydrogels, whether adhesive or not, are widely used as biomimetic scaffolds in tissue engineering applications, particularly for vascular tissue regeneration [87]. These materials provide structural support and bioactive cues that guide cell adhesion, proliferation, and differentiation. By incorporating bioactive molecules and ECM components, such as collagen, fibronectin, and laminin, hydrogels can promote the development of functional vascular networks, which are essential for engineered tissues such as artificial blood vessels and cardiac patches. The primary challenge in tissue engineering is ensuring rapid vascularization to provide nutrients and oxygen to cells. Recently, Elomaa et al. developed an innovative method to enhance vascularization in hydrogel-based tissue constructs by integrating cell sheet engineering with dynamic perfusion culture [88]. The researchers developed a vascularized scaffold by rolling a confluent human umbilical vein endothelial cell (HUVEC) sheet onto a 3D-printed, perfusable tubular scaffold. This structure was embedded in a collagen gel along with additional singularized HUVECs. Indirect co-culture with human dermal fibroblasts (HDFs) facilitated molecular crosstalk, supporting angiogenesis without inducing excessive collagen remodeling. The embedded endothelial cells self-organized and formed a dense lumen-containing 3D vascular network within three weeks. To enhance vascularization, the system was further adapted to dynamic perfusion conditions using two independently addressable circuits [88]. Within one week of perfusion, angiogenic sprouting and microvascular network formation were observed. This hybrid approach successfully generated vascular-like networks, representing a crucial advancement in bioengineered tissue vascularization [88].

### 3.2. Current Challenges and Future Directions

#### 3.2.1. Need for Self-Healing Hydrogels

Despite their adhesive properties, many hydrogels struggle to match the mechanical strength of native vascular tissues. Blood vessels endure continuous dynamic forces, including pulsatile blood flow and pressure fluctuations. Therefore, hydrogels must possess sufficient tensile strength and elasticity to withstand these forces without rupturing or degrading prematurely. Recent research has focused on incorporating nanomaterials, such as graphene oxide, silica nanoparticles, and reinforced polymer networks, to enhance the mechanical properties of hydrogels [89]. Another key challenge is the long-term stability of vascular adhesive hydrogels. Some hydrogels degrade too quickly, failing to provide sustained support for tissue repair. Conversely, slow-degrading hydrogels may lead to inflammation or fibrosis due to prolonged foreign body presence [90]. Fine-tuning the degradation rate to match the tissue regeneration process is crucial. Strategies such as crosslinking modifications, enzyme-sensitive linkages, and hybrid hydrogels with controlled degradation profiles are being explored to optimize stability [34,91,92]. To address mechanical and stability challenges, self-healing hydrogels capable of repairing microdamage and extending their functional lifespan have been developed [93]. These hydrogels utilize dynamic covalent bonds or supramolecular interactions, allowing them to reassemble after mechanical stress. The introduction of self-healing properties significantly improves durability, making these materials more suitable for vascular applications where maintaining mechanical integrity is critical.

#### 3.2.2. Stimuli-Responsive Hydrogels for Targeted Delivery

Efficient delivery and retention of hydrogels at the injury site remain a major challenge, as blood flow, tissue pulsation, and physiological movements can displace these hydrogels, reducing their effectiveness. Multiple studies have investigated advanced adhesion strategies inspired by nature, such as Mfps and gecko-inspired nanostructures, to enhance hydrogel retention onto the external surface of vascular tissues [94]. Additionally, stimuli-responsive hydrogels that solidify upon contact with physiological cues, such as temperature, pH, or enzymatic activity, are being developed to improve targeted delivery [95,96,97]. For example, thermo-responsive hydrogels that transition from liquid to gel at body temperature may have promising vascular applications. Poly (N-isopropylacrylamide) (PNIPAM)-based hydrogels are particularly well-researched due to their reversible volume changes in response to temperature variations. Below their critical temperature, PNIPAM remains hydrophilic, adopting a hydrated coil structure in aqueous environments and remaining in a swollen hydrogel state. However, when the temperature exceeds 32 °C, hydrogen bonding with water is disrupted, causing the polymer to dehydrate, resulting in substantial water expulsion and hydrogel shrinkage [98]. This reversible process enables the fabrication of microstructures, such as blood vessels smaller than 100 μm. Li et al. successfully employed cytocompatible PNIPAM-GelMA scaffolds to create microscale vasculature, achieving a minimum vessel diameter of 70 μm by using sacrificial alginate fibers [99]. Such advancements demonstrate the potential of stimuli-responsive hydrogels with added precision and effectiveness of vascular repairs.

#### 3.2.3. Minimizing Immune Response

The immune response to vascular adhesive hydrogels is another critical consideration. Although multiple hydrogels are designed to be biocompatible and biodegradable, certain formulations may still trigger chronic inflammation or foreign body reactions. One promising strategy to mitigate adverse immune responses is the incorporation of immunomodulatory agents, such as anti-inflammatory peptides or cytokine inhibitors, to regulate inflammation [100]. Furthermore, surface modifications that mimic the ECM, such as coating with ECM biomolecules or PEG, can enhance cellular integration while preventing protein adsorption and immune activation.

Integrating bioactive peptides and growth factors into hydrogel formulations can further enhance biocompatibility and therapeutic outcomes. These molecules promote angiogenesis, cell migration, and tissue regeneration, accelerating wound healing and vascular repair [101]. Advances in controlled-release systems allow the sustained delivery of these bioactive agents, improving therapeutic outcomes. Moreover, enzyme-sensitive linkages allow synchronized tissue regeneration while preventing chronic immune reactions [102]. Incorporating nanomaterials, such as graphene oxide or silver nanoparticles, can further reduce bacterial infections and modulate macrophage behavior toward tissue repair [103]. These combined strategies improve hydrogel safety profiles while also providing added clinical translation for applications, such as effectiveness for applications in drug delivery, wound healing, and tissue engineering.

#### 3.2.4. Newer Methods of Fabrication

While there has been significant progress in biomaterial fabrication for tissue adhesive hemostatic agents, there is still room for significant improvements and advancements in this field. Composite hydrogels that combine the benefits of different materials are being explored to overcome the limitations of traditional hydrogels. Hybrid hydrogels incorporating synthetic polymers, natural biopolymers, and inorganic nanomaterials have the potential to achieve superior mechanical strength, biocompatibility, and controlled degradation [104]. For instance, incorporating nanocellulose or carbon-based nanomaterials is postulated to enhance structural integrity while maintaining bioactivity [105].

A more recent and exciting development in hydrogel fabrication is 3D bioprinting, which enables the precise design of hydrogels with desirable architectures [106]. This technology enables the fabrication of patient-specific vascular grafts and tissue scaffolds with controlled porosity, mechanical properties, and bioactive functionalities. By combining 3D bioprinting with bioinks containing endothelial cells and ECM components, investigators may have an opportunity to further personalize and tailor vascular repair solutions. Unique conduits can be developed for difficult-to-reconstruct areas of the body, such as the face, appendages (figures/toes), and densely vascularized tissue and internal organs.

Another novel approach to fabricating hydrogels with intricate 3D shapes involves introducing polyelectrolytes into hydrogel precursor solutions to modulate spatial internal stresses. Incorporating polyelectrolytes enhances volume shrinkage during polymerization, allowing precise modulation of internal stresses within the hydrogel network. The resulting hydrogels exhibit the ability to undergo complex deformations in response to environmental stimuli, showcasing potential not only in soft actuators but also in opening new avenues in vascular tissue engineering and related biomedical applications. Hydrogels with tailored internal stresses can be designed to mimic the mechanical properties of vascular tissues, facilitating the development of scaffolds that support the growth and organization of endothelial cells, which is essential for creating functional blood vessels [107].

Additionally, advances in microfluidic techniques and lithographic fabrication are also facilitating the creation of hydrogel-based vascular structures with highly controlled architectures [108]. These innovations are essential for developing next-generation vascular adhesive hydrogels capable of seamlessly integrating with native tissues.

## 4. Conclusions

Adhesive hydrogels have emerged as a groundbreaking innovation in biomedical engineering, providing potential solutions for hemostasis, vascular repair, and tissue regeneration. These hydrogels offer several advantages over traditional wound closure methods, such as sutures and staples, by enabling strong adhesion to wet and dynamic biological tissues, minimizing surgical trauma, and promoting faster healing. Advances in both natural and synthetic hydrogel formulations have significantly improved their mechanical properties, biocompatibility, and functionality, expanding their applications in vascular anastomosis, hemorrhage control, and tissue engineering.

Despite these advancements, several challenges remain. Achieving optimal mechanical strength and long-term stability is critical, as vascular tissues endure constant mechanical stress and pulsatile blood flow. Additionally, controlling degradation rates to match the healing process while minimizing immune responses is crucial for ensuring safe and effective clinical applications. Another key challenge is enhancing targeted delivery and retention of hydrogels at injury sites, even under physiological movements and blood flow. Future research should focus on refining hydrogel formulations to achieve an optimal balance of adhesion, durability, and biocompatibility while ensuring ease of application in surgical settings. Integrating cutting-edge technologies, such as engineered multi-functional recombinant proteins, bioactive molecules, and 3D bioprinting, could further enhance the potential of adhesive hydrogels for a wide range of biomedical applications (Table 2).

## Figures and Tables

**Table 1:** Characteristics of different vascular adhesive materials.

Material Type	Properties	Advantages	Disadvantages	References
**Fibrin-based**	Biocompatible, promotes clotting	Widely used, supports clotting	Weak adhesion, poor durability	[19,20]
**Silk Fibroin-based**	Strong, tunable mechanical properties	High biocompatibility, robust adhesion	Poor adhesion to wet tissues, requires modification	[21,22]
**Gelatin-based**	Biocompatible, tunable adhesion	Good for tissue integration, tunable adhesion	Limited mechanical strength, processing complexity	[23,24]
**Polysaccharide-based**	Biodegradable, hemostatic, antibacterial	Promotes healing, antibacterial properties	Water absorption issues, processing challenges	[25,26]
**Polyurethane-based**	Flexible, high mechanical strength	Durable, used in vascular applications	Requires precise application, potential biocompatibility issues	[27,28]
**Polyacrylic Acid-based**	Swells in response to pH changes	Responsive to biological environments	Weak adhesion to tissues, swelling control needed	[29,30]
**Polyethylene Glycol (PEG)-based**	Hydrophilic, crosslinking for adhesion	Strong adhesion, FDA-approved versions available	Potential cytotoxicity, adhesion strength variability	[16,28]
**Recombinant Protein-based**	Bioengineered, highly tunable	Highly customizable, biomimetic adhesion	High production cost	[31,32]

**Table 2:** Existing efforts and future directions in engineering vascular adhesives of desirable properties.

	Accomplished	Challenges	References
**Rapid Adhesion**	Use of mussel-inspired catechol chemistry, fibrin-based adhesives for rapid clot formation, and photopolymerizable hydrogels for fast adhesion.	Further optimization for rapid solidification under physiological conditions; improved adhesion under dynamic vascular environments.	[22,23,63,74,75]
**Tunable Mechanical Properties**	Integration of nanomaterials like graphene oxide and silica nanoparticles; hybrid hydrogels developed to enhance flexibility and elasticity.	Precise control over mechanical tuning for different vascular sites; further integration of self-healing mechanisms.	[27,28,30,32,51,65]
**Biofunctionalization**	Incorporation of bioactive molecules, such as vascular endothelial growth factor (VEGF) and platelet-derived growth factor (PDGF), for enhanced healing.	Long-term bioactivity validation; optimizing controlled release mechanisms for sustained therapeutic effects.	[83,85,86,88,109]
**Biocompatibility**	Use of natural polymers (fibrin, silk fibroin, chitosan, gelatin) and synthetic hydrogels (PEG, polyurethane) with validated biocompatibility studies.	Addressing immune response and inflammation concerns in long-term applications; minimizing foreign body reactions.	[20,23,26,36,47]
**Sufficient Mechanical Strength**	Development of reinforced polymer networks, multi-functional protein adhesives, and high-strength hydrogel formulations to improve durability.	Achieving physiological mechanical properties of native vascular tissues; balancing strength with flexibility.	[57,69,91,92,93]
